# Mitochondrial Function in Healthy Human White Adipose Tissue: A Narrative Review

**DOI:** 10.3390/nu15204430

**Published:** 2023-10-19

**Authors:** Lisa Guerrier, Corinne Malpuech-Brugère, Ruddy Richard, Julianne Touron

**Affiliations:** 1 Unité de Nutrition Humaine, Université Clermont Auvergne, INRAe, 63000 Clermont-Ferrand, France; corinne.malpuech-brugere@uca.fr (C.M.-B.); ruddy.richard@uca.fr (R.R.); julianne.touron@gmail.com (J.T.); 2 CRNH Auvergne, 63000 Clermont-Ferrand, France; 3 CHU Clermont-Ferrand, 63000 Clermont-Ferrand, France

**Keywords:** mitochondria, white adipose tissue, human, healthy, body composition

## Abstract

As ¾ of the global population either have excess or insufficient fat, it has become increasingly critical to understand the functions and dysfunctions of adipose tissue (AT). AT serves as a key organ in energy metabolism, and recently, attention has been focused on white AT, particularly its mitochondria, as the literature evidence links their functions to adiposity. This narrative review provides an overview of mitochondrial functionality in human white AT. Firstly, it is noteworthy that the two primary AT depots, subcutaneous AT (scAT) and visceral AT (vAT), exhibit differences in mitochondrial density and activity. Notably, vAT tends to have a higher mitochondrial activity compared to scAT. Subsequently, studies have unveiled a negative correlation between mitochondrial activity and body mass index (BMI), indicating that obesity is associated with a lower mitochondrial function. While the impact of exercise on AT mitochondria remains uncertain, dietary interventions have demonstrated varying effects on AT mitochondria. This variability holds promise for the modulation of AT mitochondrial activity. In summary, AT mitochondria exert a significant influence on health outcomes and can be influenced by factors such as obesity and dietary interventions. Understanding the mechanisms underlying these responses can offer potential insights into managing conditions related to AT and overall health.

## 1. Introduction

The human body can be schematized as a physiological model with two sections: fat-free mass (water, organs, bones and muscles) and fat mass (adipose tissue (AT)). AT typically accounts for 20 to 30% of the total body mass [1]. Globally, three quarters of the population falls into either the category of having excess fat mass or insufficient fat mass. It is well established that being overweight or obese is associated with an increase in induced adverse long-term health effects such as cardiovascular diseases [2], diabetes [3], cancer, etc. [4]. During obesity, white AT can by dysfunctional because of an energy excess. This will result in ectopic fat depots in other organs, leading to insulin resistance. The phenomenon is known as “lipotoxicity” [3]. Conversely, having insufficient fat mass can also be a cause for concern, especially when it is associated with chronic diseases like cancer [5]. Cachexia, a condition resulting from certain diseases, is characterized by a reduction in muscle mass and function with or without fat mass [6]. This condition poses a significant public health challenge and leads to numerous deaths each year [7]. For instance, cancer cachexia is frequently accompanied by a depletion in AT due to increased lipolysis [8,9]. It is believed that fat loss precedes muscle loss in cancer cachexia, both contributing to a poor quality of life [10] and worsening the prognosis for certain types of cancer [5]. Therefore, gaining a deeper understanding of the mechanisms underlying the functions of AT is essential for maintaining health, whether in cases of excess fat mass or insufficient fat mass.

This tissue is mainly composed of adipocytes and pre-adipocytes, but also immune cells and fibroblasts, and its vascularization is relatively poor compared to other organs. Therefore, AT has immune, endocrine, mechanic and thermic functions [11], but is commonly known as a key organ for energy metabolism. It plays a vital role in maintaining energy balance through lipogenesis (which involves the synthesis of triglycerides) alongside the liver, muscles, heart and pancreas [12]. Additionally, it acts as the primary site for energy storage in the form of triglycerides and can release them for the energy needs of peripheral organs through lipolysis. In white adipocytes, the large lipid vacuole functions as an energy reservoir. Notably, these adipocytes possess a relatively low mitochondrial density, despite their central role in energy production and utilization [11]. This particular characteristic has led them to being under-investigated. Recent advancements in more sensitive techniques have facilitated investigations into white AT mitochondria. It is now possible to measure the mitochondrial function through quantification of oxygen consumption using high-resolution respirometry devices in cells with a low mitochondrial density, such as adipocytes. Assessing this parameter across various respiratory states, by adding substrates or inhibitors, provides insights into mitochondrial oxidative phosphorylation (OXPHOS) capacities [13]. Indeed, the energy produced by mitochondria plays a crucial role in numerous pathways, including energy metabolism, apoptosis, autophagy, and inflammation [14]. Therefore, mitochondrial dysfunction may be linked to the ATs mentioned earlier. Consequently, investigating the function and adaptability of adipocyte mitochondria could be a promising avenue for further research aimed at improving the care of patients with these conditions.

This narrative review provides an overview of mitochondrial functionality in human white AT. It begins by examining the mitochondrial function in AT based on its anatomical location, as different AT depots are known to exhibit slight variations in their functions and activities. Subsequently, the review delves into the relationship between mitochondrial activity and body composition, particularly in relation to body mass index (BMI). Finally, the review discusses interventional studies involving exercise training and dietary interventions and their impact on mitochondrial function.

The focus of this review is exclusively on clinical studies involving humans who do not have major pathologies. These studies provide data on the measurements of mitochondrial functionality in white adipose tissue biopsies or offer insights into energy expenditure and fat oxidation.

## 2. Mitochondria and White Adipose Tissue Depot Locations

AT is subdivided into depots that can be regarded as mini organs due to their distinct and specialized functions [15]. Among these, two are primarily described in the literature: subcutaneous adipose tissue (scAT) and visceral adipose tissue (vAT). As individuals age and based on their biological gender, the distribution of AT within these depots differs [16]. However, there is a notable scarcity in the literature regarding the relationship between mitochondria and gender [17,18,19] or age [17,20].

**Figure 1:**
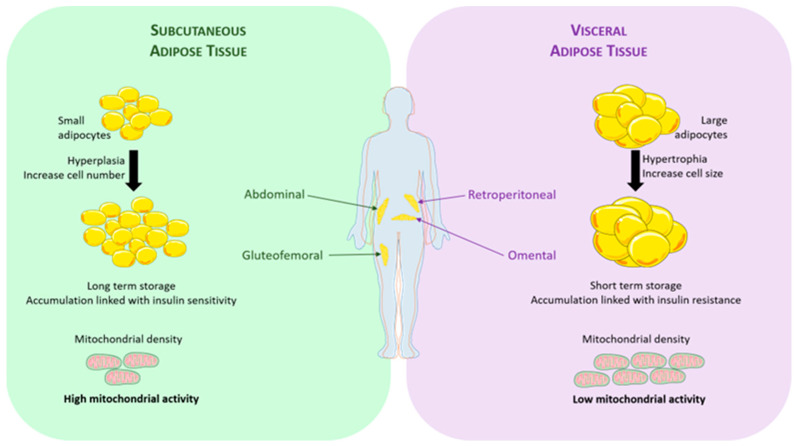
White adipose tissue characteristics according to their location.

ScAT is predominantly located in the abdominal and gluteo-femoral regions as depicted in Figure 1. This type of AT is characterized by the presence of small adipocytes [21]. It can grow by both increasing the number of adipocytes (hyperplasia) and enlarging their size (hypertrophy) [22]. ScAT serves as a reservoir of triglycerides to meet long-term energy demands. Notably, its accumulation is associated with improved insulin sensitivity [23].

VAT can constitute a significant portion of total body fat, accounting for up to 40% of the overall fat mass. It is distributed between the omental and retro-peritoneal regions, as depicted in Figure 1 and summarized in Table 1, and exerts a substantial metabolic impact on health. VAT is characterized by the presence of large adipocytes [21], and its expansion primarily occurs through hypertrophy, with limitations imposed by its anatomical location [22]. Due to its relatively lower storage capacity compared to scAT, lipids must be redistributed to liver and muscles, which can lead to insulin resistance in these tissues [24]. Indeed, studies have consistently indicated that the accumulation of vAT, but not scAT, poses a high risk for the development of insulin resistance and type 2 diabetes (T2D) [23].

When focusing on mitochondria, studies have compared their functionality between adipose depots. VAT has been found to have twice the concentration of mitochondria per milligram of tissue as scAT, with counts of 12.1 ± 1.2 × 10^6^ ds mtDNA/mg tissue in vAT compared to 6.5 ± 0.8 × 10^6^ ds mtDNA/mg tissue in scAT [21]. These measurements were conducted in a group of 20 obese patients (BMI 40.7 ± 1.3 kg·m^−2^), consisting of 16 women and 4 men, who were eligible for bariatric surgery. Additionally, the mitochondrial function was evaluated to be significantly more important in scAT than in vAT when normalized to mitochondrial DNA content. In brief, although scAT has fewer mitochondria, they exhibit a higher activity compared to those in vAT. Another study focused on evaluating differences between subcutaneous abdominal and gluteal depots in black South African women with a BMI ranging from 30 to 40 kg·m^−2^ [23]. This study concurrently measured mitochondrial function and H_2_O_2_ production, which is a marker related to reactive oxygen species (ROS) production. Results revealed that the electron capacity was greater in gluteal AT than in abdominal AT, but no differences were observed in other mitochondrial parameters (i.e., mitochondrial respiration, mitochondrial content). However, H_2_O_2_ production was significantly higher in gluteal AT than in abdominal AT under all measured conditions. The increased activity of the electron transport system in gluteal AT could suggest an uncoupling between oxidation and phosphorylation, leading to the observed higher ROS production observed. Nonetheless, the study did not report quantification of uncoupling proteins (UCP) or antioxidant defenses like superoxide dismutase (SOD), which can modulate ROS production and its effects. In a third study involving 153 patients undergoing abdominal surgery, both lean and obese subjects showed that *pgc-1α* (*peroxisome proliferator-activated receptor gamma coactivator-1 alpha*), a marker for mitochondrial biogenesis, was more expressed in scAT than in vAT [25]. In a proteomic study that compared human adipocytes in vAT, abdominal scAT and gluteo-femoral scAT, the population consisted of five obese women (BMI 47.3 ± 1.4 kg·m^−2^) undergoing bariatric surgery after an overnight fast [26]. When examining the main proteins differentially regulated between depots, those involved in mitochondrial dysfunction ranked highest in differences between gluteal scAT and the two other depots, but they were only in the third position when comparing abdominal scAT and vAT. This suggests that proteins related to mitochondrial dysfunction exhibit more differential expression between gluteal scAT and vAT than between abdominal scAT and vAT. Furthermore, when focusing specifically on respiratory chain complex proteins, all were more highly expressed in gluteal scAT than in abdominal scAT. Most of the β-oxidation proteins studied were also more expressed in gluteal scAT compared to both abdominal scAT and vAT. In a population of 32 obese subjects, when examining markers related to browning and mitochondrial activity, vAT showed higher expression of markers such as *ucp1* (*Uncoupler Protein-1*), *cidea* (*Cell Death Inducing DFFA Like Effector A*), *prdm16* (*PR domain containing 16*), *tbx1* (*T-box transcription factor 1*) and *p2rx5* (*Purigenic receptor P2X 5*) compared to scAT. Additionally, mitochondrial markers like *pparγ* (*peroxisome proliferator-activated receptor γ*), *atp5a* (*mitochondrial membrane ATP synthase*) and *ndufa1* (*NADH:Ubiquinone oxidoreductase subunit A1*) are more expressed in vAT than in scAT [27]. Consequently, vAT demonstrates a metabolic activity that is more favorable for browning, as well as exhibiting greater mitochondrial biogenesis and activity when compared to scAT.

AT displays a specific mitochondrial metabolism that can vary depending on the specific depots within the same individual. Furthermore, differences in mitochondrial metabolism have also been observed between individuals, particularly in relation to their body composition. These variations highlight the complexity of AT and its adaptative responses to both anatomical location and individual characteristics. Understanding these differences is crucial for unraveling the intricate relationship between AT and metabolic health.

## 3. White Adipose Tissue Mitochondria and Body Composition

Researchers have demonstrated a keen interest in white AT mitochondria in individuals with both a low BMI (<18.5 kg·m^−2^) and a high BMI (>30 kg·m^−2^). Investigations have revealed differential mitochondrial activities based on body composition using various methods including respirometry, enzymatic assays, protein analysis and transcriptomic analysis. This comprehensive approach allows for a more thorough understanding of how AT mitochondria respond to variations in body composition.

When examining the relationship between mitochondrial analyses and BMI, two major studies reached the same conclusion: there exists a negative correlation between mitochondrial respiration and BMI [28,29]. The first study investigated mitochondrial respiration in subcutaneous adipocytes of 16 women who were referred for abdominal surgery and had BMIs ranging from 18 to 36 kg·m^−2^ [28]. In this study, it was observed that obese patients exhibited a significant reduction in the levels of complex I (NDUFB8) and complex IV (MTCO2) proteins compared to non-obese individuals. The second study included 47 men and women referred for abdominal laparoscopic surgery who had BMIs ranging from 21 to 70 kg·m^−2^. This study examined both scAT and vAT [29]. It was found that the correlation between mitochondrial respiration and BMI was significant only in scAT. Furthermore, a negative correlation between BMI and mitochondrial content was also reported by Kaaman et al. in a cohort consisting of 116 women (72 obese with a BMI of 37 ± 5 kg·m^−2^ and 44 non-obese with a BMI of 24 ± 3 kg·m^−2^) and 32 men (23 obese with a BMI of 36 ± 5 kg·m^−2^ and 9 non-obese with a BMI of 27 ± 2 kg·m^−2^) [17]. In obese participants, Wessels et al. concluded that an increased weight had a more significant impact on decreasing mitochondrial respiration than glycemic status [29]. This finding was further supported by another study involving women undergoing abdominal surgery, which examined the size of adipocytes and their potential relationship with citrate synthase activity and high-resolution respirometry [30]. According to their results, obesity itself led to a decrease in the oxygen consumption rate and global mitochondrial dysfunction. Two additional studies confirmed a decrease in citrate synthase activity in the mitochondria of obese individuals compared to non-obese subjects. The first study involved 135 adult men and women: 90 obese (BMI 42.3 ± 7.0 kg·m^−2^) who underwent bariatric surgery and 45 non-obese (24.7 ± 3.0 kg·m^−2^) who underwent elective surgery [31]. In the second, 10 lean or obese male children aged 8 to 12 years old underwent appendicitis surgery [32]. These consistent findings emphasize the negative association between BMI and mitochondrial function, highlighting the impact of obesity on AT mitochondria.

The dysregulation observed in the mitochondria of obese individuals appears to be reversible, as suggested by several studies. One study showed an improvement in mitochondrial maximal capacity following a Roux-en-Y gastric bypass surgery, leading to a substantial weight loss of 40 ± 2 kg in morbidly obese patients [33]. This improvement suggests that the dysregulation observed in mitochondria can be reversed with significant weight loss. Van Der Kolk et al. investigated the effects of weight loss following the same bariatric intervention in a cohort of 172 obese men and women (BMI of 43.0 ± 5.2 kg·m^−2^) [34]. Through differential RNA expression analyses, they observed an increase in OXPHOS, TCA (tricarboxylic acid) cycle and fatty acid β-oxidation-related genes 12 months post-surgery (BMI 33.3 ± 5.0 kg·m^−2^). In patients with obesity and insulin resistance, weight loss following a bariatric surgery increased the levels of scAT mitochondrial proteins involved in its architecture (mitofilin) and biogenesis (*pgc1α*) [35]. However, the opposite effect was observed in obese patients with normoglycemic status. A study comparing 26 monozygotic twins (9 men and 17 women), where one twin was obese (BMI 31.3 ± 1.0 kg·m^−2^) and the other was not (BMI 25.3 ± 0.9 kg·m^−2^), found that the OXPHOS pathway in abdominal scAT was significantly downregulated in obese twins [36]. The mitochondrial content, as measured by the mitochondrial DNA/genomic DNA ratio, was also decreased in obese subjects compared to their lean twin. This suggests that mitochondrial differences are more related to lifestyle factors than genetic factors and can potentially be reversed. In a study involving omental vAT obtained from 19 women and 4 men with obesity (BMI 54.6 ± 2.0 kg·m^−2^) undergoing bariatric surgery, transcriptomic analyses revealed that mitochondrial genes were upregulated after post-operative weight loss, indicating a restoration of mitochondrial metabolism [37]. These findings collectively suggest that weight loss and lifestyle changes can have a positive impact on mitochondrial function and gene expression in AT, even in individuals with obesity-related metabolic dysregulation.

Unlike obesity, thinness, characterized by a low BMI (≤18.5 kg·m^−2^), is often associated with underlying medical conditions (i.e., eating disorders, cachexia). However, there is a non-pathological condition known as constitutional thinness (CT), where mitochondrial metabolism can be studied in healthy individuals [38]. In a study involving 29 CT subjects, bioenergetic parameters were compared with those of 29 control subjects after challenging them with overfeeding. The study found that mitochondrial respiration (Complex II) was more important in subjects with CT compared to normal-weight controls, and this effect was consistent across both sexes. Additionally, CT subjects were found to have smaller fat cells compared to controls but exhibited a higher mitochondrial density. The summarized studies are presented in Table 2, which provides a comprehensive overview of the research in this area.

Based on the literature, there is a clear link between mitochondrial functionality in white AT and adiposity, with mitochondrial function being negatively correlated with BMI. However, this phenomenon appears to be reversible with the implementation of weight loss strategies.

## 4. White Adipose Tissue Mitochondria and “Interventions”

A more comprehensive understanding of how to modulate AT mitochondrial activity and the effective interventions could provide valuable insights into combating AT disorders. To date, the literature is relatively scarce, but some clinical studies have already begun to explore the impact of exercise and nutritional interventions on mitochondrial function and AT metabolism. These studies represent important steps towards developing strategies to improve mitochondrial health in AT and address AT-related disorders.

### 4.1. Exercice Training

The impact of exercise on mitochondrial activity in white AT has been less studied compared to its well-documented effects on skeletal muscle [39,40,41]. The results from existing research in this area are still a subject of debate, and there is no consensus.

Some studies have compared sedentary individuals to lifelong trained populations [20,42]. One study focused on 12 lifelong exercise-trained (2 h of endurance exercise training per week from late adolescence) men and 10 untrained men aged 62 to 73 years old [20]. The scAT mitochondrial function and contents were found to be higher in the physically active population compared to the sedentary group. Another study involved 9 sedentary lean and overweight subjects (3 men and 6 women) and 7 active (4 cumulative hours of moderate to vigorous aerobic training at least 3 times a week) men [42]. This study concluded that exercise training (aerobic training for 3 weeks) alone was not sufficient to reverse the effects of a sedentary lifestyle on the mitochondrial function.

When examining the effects of exercise training on the mitochondrial function, the results from various studies are discordant. Some studies found no significant effect of exercise training on AT mitochondria [23,39,42,43,44,45,46], while another reported a decrease in mitochondrial respiratory capacities after training [47]. Some studies also observed an increase in mitochondrial content, biogenesis [25,44,48] and mitochondrial respiration [49].

The inconsistent findings regarding the impact of exercise on the AT mitochondrial function suggest that the relationship between exercise and white AT mitochondria is complex. The summarized results of these studies, presented in Table 3, provide an overview of the research in this area. Indeed, there are multiple factors contributing to the variability in the effects of exercise on white AT mitochondria, and this complexity makes it challenging to draw conclusions. Exercise programs can vary widely in terms of duration, intensity and type (i.e., endurance versus strength training). Just as different types of exercise have distinct effects on skeletal muscle [50,51], it is reasonable to assume that they may also have varying impact on AT. On the other hand, study populations often exhibit significant heterogeneity. While they are generally categorized as sedentary, differences in sex, age and BMI within and between studies can influence the outcomes, which may contribute to the mixed findings in the literature. Finally, participants can respond differently to exercise due to genetic, physiological and lifestyle differences. This individual variability further complicates efforts to establish consistent patterns in AT mitochondrial responses to exercise.

### 4.2. Diet

There is relatively limited research on the impact of nutritional interventions, including total energy intake, macronutrient composition and natural bioactive compounds, on AT mitochondrial activity modulation.

The impact of different dietary interventions on mitochondrial activity in AT has been explored in a few studies. In one study involving overweight/obese subjects (25 men and 28 women), both a 12-week low calorie diet (1250 kcal/day) and a 5-week very low calorie diet (500 kcal/day) led to downregulation of OXPHOS and TCA cycle genes in scAT biopsies [53]. This downregulation was more important in the very-low-calorie diet group. A similar impact of a very-low-calorie diet (500 to 600 kcal/day) on mitochondrial gene expression was observed in skeletal muscle in 9 obese women treated with this diet for 53 days [54]. Another clinical study compared 23 obese (BMI 36 ± 5 kg·m^−2^) and 9 non-obese men (BMI 27 ± 2 kg·m^−2^) and 72 obese women (BMI 37 ± 5 kg·m^−2^) and 44 non-obese women (BMI 24 ± 3 kg·m^−2^) undergoing high-fat or low-fat diets [17]. Regardless of the diet type, no difference was found in mitochondrial DNA copy numbers. A study compared a long-term self-adherence ketogenic diet (n = 5) against a standard American diet (control group) (n = 4) in young men and women with a normal BMI. The ketogenic diet group exhibited a higher mitochondrial respiration in scAT, measured by oxygraphy [55]. Interestingly, this increased respiration was not associated with a higher production of ATP, suggesting mitochondrial uncoupling and the dissipation of energy as heat. The study suggested that ketones may stimulate energy wasting in human adipocytes [55]. In a study by Bikman et al., a 20-week high-carbohydrate diet (60% of total energy) was examined in 10 overweight men and women after weight loss (11.7 ± 2.0%) through caloric restriction (diet at 60% of energy requirements) [56]. This study found that the high carbohydrate diet led to a reduction in the abdominal scAT mitochondrial function. The authors suggested that this diet might favor fat storage over oxidation, potentially contributing to weight gain. Another study focused on mitochondrial gene expression in overweight and obese men and women (BMI 27–45 kg·m^−2^) after a 2-month low calorie diet (800 kcal/day) [34]. Following weight loss, OXPHOS-related genes as well as TCA cycle- and fatty acid β-oxidation-related genes were downregulated. Jokinen et al. conducted a study comparing individuals who continuously lost weight during a 12-month low-calorie diet with those who regained weight after initial loss [57]. The group that continuously lost weight exhibited a higher expression of OXPHOS and TCA cycle proteins compared to the group that regained weight. These findings highlight the sensitivity of AT mitochondria to dietary changes and weight loss.

Several studies have explored the potential effects of natural compounds and supplements on mitochondrial metabolism and fat oxidation in AT. Venables et al. investigated the supplementation of green tea extracts in combination with cycling exercise in 12 healthy and normal-weight men (age: 26 ± 2 years and BMI 23.9 ± 0.8 kg·m^−2^) [58]. The study found that ingestion of green tea extracts one hour before exercise increased fat oxidation compared to a control group that ingested a placebo. While this study did not directly measure mitochondrial functionality, it suggests that green tea extracts may have a modulatory effect on mitochondrial metabolism. A study involving 10 healthy men (age 25 ± 1 years and BMI 25.1 ± 1.2 kg·m^−2^) measured the energy expenditure and substrate oxidation after green tea extract ingestion [59]. The results showed that fat oxidation was increased following the ingestion of green tea extracts. While the two previous studies did not specifically measure mitochondrial function, they offer insights into the potential effects of natural compounds on fat oxidation and substrate metabolism in AT. Another study focused on taurine supplementation in 8 obese women (BMI: 34.1 ± 1.2 kg·m^−2^) [49]. This supplementation resulted in an increase in fatty acid oxidation genes in scAT, although it had no effect on mitochondrial respiration. All the studies are summarized in Table 4.

Understanding how different aspects of nutrition affect AT mitochondria could provide valuable insights into metabolic health and the development of strategies to address AT-related disorders. Future research in this area may yield important findings; there is still much to explore regarding the interplay between nutrition and AT mitochondria.

## 5. Conclusions

White AT plays a central role in energy metabolism despite its relatively low mitochondrial density. The existing literature suggests a potential link between mitochondrial activity in AT and adiposity. A more profound comprehension of these mechanisms could lead to improved understanding and management of patients with adipose-related disorders.

One key insight from the available research is that different depots of white AT exhibit variations in terms of mitochondrial activity, density and function. Notably, vAT has a higher mitochondrial density and a greater level of browning proteins compared to scAT.

Multiple studies have now well documented a negative correlation between mitochondrial activity and BMI. Obesity is associated with a reduction in mitochondrial oxygen consumption, which can be reversed through weight loss.

The impact of exercise training on modulating mitochondrial activity remains a subject of debate, with no definitive conclusions reached. Future research in this area could benefit from more standardized exercise interventions, larger and more homogenous study populations and consideration of individual variability. Such approaches could help clarify the specific effects of different types of exercise on AT mitochondria and provide a clearer understanding of how exercise can be used as a tool to modulate AT function.

Regarding dietary interventions, only a limited number of clinical studies have been published. It is imperative to conduct human studies involving supplementation with various bioactive compounds to determine their effects. Different dietary interventions can have varying effects on mitochondrial gene expression and function, and the outcomes may depend on factors such as the duration and composition of the diet. These findings highlight the complex relationship between diet and mitochondrial activity in AT. Understanding these effects is important for gaining insights into how diet can influence energy metabolism and overall metabolic health. It may also be of interest to combine both exercise training and dietary intervention to maximize their effects and enhance their impact on mitochondrial functionality.

## Figures and Tables

**Table 1:** Mitochondrial function according to adipose tissue location.

Reference	Adipose Tissue Location	Population Characteristics	Mitochondrial Analysis	Tissue Comparison
Kraunsøe (2010) [21]	Visceral (*omentum majus*) vs. subcutaneous abdominal	4 men and 16 women aged 41.1 ± 2.0 years BMI: 40.7 ± 1.3 kg·m^−2^	Mitochondrial density	vAT > scAT
			Mitochondrial respiration	scAT > vAT
Mendham (2020) [23]	Subcutaneous abdominal vs. subcutaneous gluteal	35 black south African women aged 20–35 years BMI: 30–40 kg·m^−2^	Mitochondrial respiration	x
			H_2_O_2_ production	glut scAT > abd scAT
Ruschke (2010) [25]	Visceral vs. subcutaneous abdominal	Lean group 28 men and 30 women aged 50.2 ± 15.7 years BMI: 23.9 ± 1.4 kg·m^−2^	Mitochondrial biogenesis	scAT > vAT
		SC obese 28 men and 30 women aged 55.3 ± 11.6 years BMI: 33.6 ± 6.8 kg·m^−2^		
		Visceral obese 19 men and 18 women aged 64.4 ± 13.2 years BMI: 33.6 ± 6.1 kg·m^−2^		
Raajendiran (2021) [26]	Visceral vs. subcutaneous abdominal vs. gluteal subcutaneous	5 women BMI: 47.3 ± 1.4 kg·m^−2^	Mitochondrial dysfunction	abd scAT > vAT glut scAT > vAT glut scAT > abd scAT
Zuriaga (2017) [27]	Visceral omental vs. subcutaneous abdominal	8 men and 24 women aged 42 ± 2 years BMI: 43 ± 1 kg·m^−2^	Browning markers	vAT > scAT

Results are presented either as ranges or as means ± S.E.M. abd: abdominal; BMI: body mass index; glut: gluteal; H_2_O_2_: hydrogen peroxide; scAT: subcutaneous adipose tissue; vAT: visceral adipose tissue; vs.: versus.

**Table 2:** Mitochondrial function according to body composition.

Reference	Adipose Tissue Location	Population Characteristics	Mitochondrial Analysis	Mitochondrial Effects
Fischer (2015) [28]	Subcutaneous abdominal	16 women aged 41 ± 13 years BMI: 18 to 36 kg·m^−2^	Mitochondrial respiration	↓ when BMI ↑
Wessels (2019) [29]	Subcutaneous abdominal and visceral	27 women and 13 men aged 46 ± 13 years BMI: 21 to 70 kg·m^−2^	Mitochondrial respiration	↓ when BMI ↑ in scAT
Kaaman (2007) [17]	Subcutaneous abdominal	116 women and 32 men aged 39 ± 9 years BMI: 33 ± 7 kg·m^−2^	Mitochondrial density	↓ when BMI ↑
Yin (2014) [30]	Subcutaneous abdominal and visceral	20 women and 19 men aged 25 to 75 years BMI: 17.8 to 58.0 kg·m^−2^	Mitochondrial respiration	↓ when BMI ↑
			Mitochondrial density	↓ when BMI ↑
Christe (2013) [31]	Visceral	90 obese aged 48.7 ± 12.9 years BMI: 42.3 ± 7.0 kg·m^−2^ 45 non-obese aged 62.8 ± 13.9 years BMI: 24.7 ± 3.0 kg·m^−2^	Mitochondrial enzymatic activity	↓ when BMI ↑
Zamora-Mendoza (2018) [32]	Subcutaneous abdominal	5 lean boys Aged 10.0 ± 1.4 years BMI: 15.6 ± 1.3 kg·m^−2^ 5 obese boys aged 10.8 ± 1.6 years BMI: 26 ± 2.8 kg·m^−2^	Mitochondrial enzymatic activity	↓ when BMI ↑
Hansen (2015) [33]	Subcutaneous abdominal	19 women and 6 men aged 38 ± 2 years BMI: 42 ± 1 kg·m^−2^	Mitochondrial respiration	↑ after weight loss
			Mitochondrial density	= after weight loss
Van Der Kolk (2021) [34]	Subcutaneous abdominal	124 women and 48 men aged 48.3 ± 9.3 years BMI: 43.0 ± 5.2 kg·m^−2^	Mitochondrial genes	↑ after weight loss
Moreno-Castellanos (2016) [35]	Subcutaneous abdominal	Normoglycemic 9 women aged 38.2 ± 92.8 years BMI: 47.8 ± 2.4 kg·m^−2^	Mitochondrial proteins	↓ after weight loss
		Insulin resistant 9 women Aged 44.1 ± 3.5 years BMI: 51.2 ± 1.7 kg·m^−2^	Mitochondrial proteins	↑ after weight loss
Heinonen (2015) [36]	Subcutaneous abdominal	17 women and 9 men Monozygotic twins aged 29.9 ± 0.9 years BMI (lean): 25.3 ± 0.9 kg·m^−2^ BMI (obese): 31.3 ± 1.0 kg·m^−2^	Mitochondrial genes	↓ in the obese twin compared to the lean co-twin
Gonzalez-Franquesa (2022) [37]	Visceral	19 women and 4 men BMI: 37.1 to 78.9 kg·m^−2^	Mitochondrial genes	↑ after weight loss
Ling (2019) [38]	Subcutaneous abdominal	Constitutional thinness 14 women and 15 men aged 25.0 ± 4.7 years BMI: 16.96 ± 0.74 kg·m^−2^ Control 15 women and 14 men aged 22.6 ± 2.9 years BMI: 22.99 ± 1.03 kg·m^−2^	Mitochondrial respiration	↑ in constitutional thinness

Results are presented either as ranges or as means ± S.E.M. BMI: body mass index; scAT: subcutaneous adipose tissue, mitochondrial parameters are less important: ↓, more important: ↑, or equal = compared to a control group or after an intervention.

**Table 3:** Effect of exercise on subcutaneous adipose tissue mitochondria.

Reference	Exercise Modalities	Adipose Tissue Location	Population Characteristics	Mitochondrial Analysis	Mitochondrial Effects
Camera (2010) [52]	10 consecutive days of alternate endurance and HIIT training Endurance exercise: Cycling at 70% VO_2_ max 60–90 min HIIT: Cycling at 90% VO_2_ max 6 × 5 min	Subcutaneous abdominal	11 healthy non-endurance trained, non-smoking men aged 21.7 ± 0.7 years BMI: 24.3 ± 1.1 kg·m^−2^	Mitochondrial enzymatic activity	X
				Browning gene expression	X
				WAT oxidative capacity	X
Ruschke (2010) [25]	Endurance exercise 60 min of supervised training 20 min biking or running 20 min swimming 20 min warming up/cooling down 3 times/week 4 weeks	Visceral and subcutaneous abdominal	60 Caucasian men and women Categorized into groups of glucose tolerance NGT: 9 men and 11 women aged 32.8 ± 11.0 years BMI: 24.3 ± 1.5 kg·m^−2^ IGT: 9 men and 11 women aged 56.0 ± 11.5 years BMI: 29.8 ± 3.9 kg·m^−2^ T2D: 11 men and 9 women aged 53.1 ± 6.7 years BMI: 31.4 ± 3.2 kg·m^−2^	Gene expression in WAT: mitochondrial biogenesis	↑
Larsen (2015) [39]	HIIT 15 min (60 s high/90 s low ×5) 3 times/week 6 weeks	Subcutaneous abdominal	10 overweight untrained men (8) and women (2) aged 38 ± 3 years Weight: 100.1 ± 5.0 kg Fat: 37.9 ± 2.6%	High resolution respirometry	X
				Citrate synthase activity	X
Pino (2016) [42]	Endurance exercise 70 to 85% VO_2_ max 30 to 60 min 6 times/week 3 weeks	Subcutaneous abdominal	9 sedentary men (3) and women (6) aged 29.33 ± 7.42 years BMI: 26.65 ± 1.97 kg·m^−2^	Mitochondrial DNA copy	X
			7 active men aged 29.33 ± 7.42 years BMImin: 26.65 ± 1.97 kg·m^−2^	Gene expression: beige adipose genes	X
Tsiloulis (2017) [45]	Endurance exercise 30 min of cycle ergometer at 75% HRmax (3 sessions/week) Interval Session 5 to 7 series: 3 min 85% HRmax/3 min 65% HRmax (1 session/week) 4 times/week 6 weeks	Subcutaneous abdominal and gluteofemoral	6 healthy overweight sedentary men aged 37.3 ± 2.3 years BMI: 30.1 ± 2.3 kg·m^−2^	Gene expression: beige adipose genes	X
Dreher (2023) [46]	Endurance exercise 1 h: 30 min of cycling and 30 min of walking on a treadmill at 80% VO_2_ max 3 times/week 8 weeks	Subcutaneous abdominal	14 healthy sedentary women (8) and men (6) aged 27.90 ± 4.11 years BMI: 31.20 ± 3.67 kg·m^−2^	High resolution respirometry	x
				Mitochondrial biogenesis	x
Dohlmann (2018) [47]	HIIT 7 bouts of 1 min intensity up to 90% VO_2_ max 3 times/week 6 weeks	Subcutaneous abdominal	12 healthy sedentary men (5) and women (7) aged 40 ± 2 years BMI: 32 ± 3 kg·m^−2^	High resolution respirometry	↓
				Mitochondrial DNA content	X
Brandao (2019) [48]	Alternating strength and endurance exercise 15 stations of 30 s (at least 10 repetitions) alternated with 30 s jogging 55 min/session 3 times/week 10 weeks	Subcutaneous abdominal	14 sedentary women aged 35 ± 6 years BMI: 33 ± 3 kg·m^−2^	High resolution respirometry	↓
				Citrate synthase activity	↑
Hoffman (2020) [43]	Strength exercise 80% VO_2_ max 30 min of walk and 30 min of bicycle 8 weeks	Subcutaneous abdominal	25 healthy sedentary men (9) and women (16) aged 29.8 ± 8.4 years BMI: 31.5 ± 4.3 kg·m^−2^	High resolution respirometry	X
Mandrup (2020) [44]	Endurance training 53 min of bike exercise with 3 blocks of intensity 3 times/week 3 months	Abdominal and femoral subcutaneous	40 pre-menopausal et 39 post-menopausal women aged 45 to 57 years BMI: 23.5 kg·m^−2^	Western blot: OXPHOS	↑
Mendham (2020) [23]	Endurance and strength training Endurance: 60–70% HR_peak_ Strength: 75–80% HR_peak_ 40 to 60 min 4 days/week 12 weeks	Subcutaneous abdominal and gluteal (liposuction)	35 sedentary black South African women aged from 20 to 35 years BMI: 30 to 40 kg·m^−2^	High resolution respirometry Mitochondrial DNA	X
De Carvalho (2021) [49]	Endurance training and strength training 15 stations of resistance exercises for 30 s, 10 times alternated with 30 s of jogging Total volume: 55 min 75 to 90% of heart rate 3 times/week 8 weeks	Subcutaneous abdominal	8 women aged 33.9 ± 1.9 years BMI: 32.4 ± 0.9 kg·m^−2^	Fatty acid oxidation gene expression	↑
				Mitochondrial respiration	↑

Results are presented either as ranges or as means ± S.E.M. BMI: body mass index; GT: glucose tolerance; HIIT: high-intensity interval training; HRmax: maximal heart rate; HR_peak_; peak heart rate; IGT: impaired glucose tolerance; NGT: normal glucose tolerance; min: minutes; OXPHOS: oxidative phosphorylation; T2D: type 2 diabetes; VO_2_ max: maximal oxygen uptake; WAT: white adipose tissue, mitochondrial parameters are less important: ↓, more important: ↑, or equal = after the training intervention.

**Table 4:** Effect of dietary intervention on adipose tissue mitochondria.

Reference	Intervention	Adipose Tissue Location	Population Characteristics	Mitochondrial Analysis	Mitochondrial Effects
Vink (2017) [53]	LCD 1250 kcal/day	Subcutaneous abdominal	14 women and 13 men aged 51.7 ± 2.1 years BMI: 31.5 ± 0.5 kg·m^−2^	OXPHOS and TCA (transcriptomic)	↑ after weight loss ↓ during weight loss
	VLCD 500 kcal/day		14 women and 12 men aged 50.4 ± 1.5 years BMI: 30.8 ± 0.4 kg·m^−2^	OXPHOS and TCA (transcriptomic)	↑ after weight loss ↓ during weight loss
Kaaman (2007) [17]	LFD or HFD	Subcutaneous abdominal	116 women and 32 men aged 39 ± 9 years BMI: 33 ± 7 kg·m^−2^	Mitochondrial DNA copy number	= after weight loss
Bikman (2022) [56]	High Carbohydrate Diet	Subcutaneous abdominal	7 women and 3 men BMI: 30.0 ± 2.9 kg·m^−2^	Mitochondrial respiration (high resolution respirometry)	↓ after weight loss
Van Der Kolk (2021) [34]	LCD 800 kcal/day	Subcutaneous abdominal	203 women and 111 men aged 42.8 ± 6.6 years BMI: 27 to 45 kg·m^−2^	OXPHOS (RNA sequencing)	↓ after weight loss
Jokinen (2017) [57]	VLCD	Subcutaneous	6 weight loss subjects BMI: 35 ± 0.7 kg·m^−2^	OXPHOS TCA (Transcriptomic)	Controls > weight losers
				Mitochondrial DNA copy number	↓ after 12 months VLCD
Venables (2008) [58]	Green tea extract supplementation	/	12 men aged 26 ± 2 years BMI: 23.9 ± 0.8 kg·m^−2^	Fat oxidation	↑ after ingestion of green tea extract
Dulloo (1999) [59]	Green tea extract and caffeine	/	10 men aged 25 ± 1 years BMI: 25.1 ± 1.2 kg·m^−2^	Energy expenditure	↑ after ingestion of green tea extract
				Fat oxidation	↓ after ingestion of green tea extract
De Carvalho (2021) [49]	Taurine	Subcutaneous abdominal	8 women aged 31.9 ± 2.1 years BMI: 34.1 ± 1.2 kg·m^−2^	Fatty acid oxidation gene expression	↑ after taurine supplementation
				Mitochondrial respiration	= after Taurine supplementation

Results are presented either as ranges or as means ± S.E.M. BMI: body mass index, HFD: high-fat diet; LCD: low-calorie Diet, LFD: low-fat diet; VLCD: very-low-calorie diet, OXPHOS: oxidative phosphorylation; TCA: tricarboxylic acid cycle, mitochondrial parameters are less important: ↓, more important: ↑, or equal = after the dietary intervention.

## Data Availability

Not applicable.
